# Aryl Hydrocarbon Receptor Alleviates Hepatic Fibrosis by Inducing Hepatic Stellate Cell Ferroptosis

**DOI:** 10.1111/jcmm.70278

**Published:** 2024-12-09

**Authors:** Shenghui Liu

**Affiliations:** ^1^ Lin He's Academician Workstation of New Medicine and Clinical Translation Jining Medical University Jining China

**Keywords:** aryl hydrocarbon receptor, ferroptosis, liver fibrosis, mouse hepatic stellate cells, multidrug‐resistant protein 1

## Abstract

Liver injury‐induced activation of hepatic stellate cells (HSCs) is a crucial step in the progression of liver fibrosis. The aryl hydrocarbon receptor (AHR), a ligand‐activated transcription factor, is highly expressed in the liver. However, the role of AHR in liver fibrosis remains controversial. Our study revealed that the nontoxic ligand YH439 directly activated the AHR and regulated the expression of multidrug‐resistant protein 1 (*Mrp1*) in mouse hepatic stellate cells (mHSCs), thereby diminishing the antioxidant capacity of mHSCs by promoting GSH efflux, and specifically inducing mHSCs ferroptosis without affecting hepatocytes. In a chronic liver fibrosis model, YH439 activated AHR to promote mHSC ferroptosis without causing hepatocyte ferroptosis, thereby alleviating liver fibrosis. Conclusively, this study shows that AHR alleviates liver fibrosis in mice by selectively inducing mHSC ferroptosis without causing hepatocyte ferroptosis and suggests that AHR is a potential target for the treatment of liver fibrosis.

## 1 Introduction

Liver fibrosis is characterized by the excessive accumulation of extracellular matrix (ECM) proteins, leading to cirrhosis, portal hypertension, liver failure, and an increased risk of hepatocellular carcinoma [1]. Activated hepatic stellate cells (HSCs) are primarily responsible for ECM protein production and the release of profibrotic cytokines [2, 3]. In a quiescent state, HSCs express desmin and glial fibrillary acidic proteins (GFAP). Upon activation, HSCs express α‐smooth muscle actin (αSMA). The activation process involves the upregulation of fibrotic genes and the proliferation of HSCs, which form the core pathogenesis of liver fibrosis [4]. The transforming growth factor‐β (TGF‐β) pathway plays a crucial role in HSC activation [5]. Thus, HSCs represent potential target cells for the treatment of liver fibrosis.

The aryl hydrocarbon receptor (AHR) is a ligand‐activated transcription factor highly expressed in the liver [6]. AHR regulates the metabolism of exogenous compounds and participates in many important physiological processes, such as the cell cycle, cell proliferation, and regulation of the immune response [7]. Different ligands may have different effects on AHR activation [8, 9]. AHR activated by 2,3,7,8‐tetrachlorodibenzo‐*p*‐dioxin (TCDD) can cause hepatotoxicity and activate HSCs, leading to liver fibrosis. Conversely, the nontoxic endogenous ligand 2‐(1H‐indole‐3‐carbonyl)‐thiazole‐4‐carboxylic acid methyl ester (ITE) activates AHR, which inhibits HSC activation and inhibit liver fibrosis in mice [8]. The development of nontoxic AHR agonists and the investigation of their mechanisms of action may have positive implications for the prevention or treatment of liver fibrosis.

Multidrug‐resistant protein 1 (MRP1) is a member of the ATP‐binding cassette (ABC) transporter family [10]. MRP1 is commonly found in the lungs, testicles, kidneys, bones, heart muscle, and placental tissues. MRP1 is not expressed in normal hepatocytes but is highly expressed by immortalized hepatocytes, hepatocellular carcinoma cells, and HSCs [11, 12]. The transport process of MRP1 relies on ATP consumption and is thus closely associated with ATP [13, 14]. MRP1 cannot independently transport unmodified exogenous compounds but can effectively transport conjugated complexes after binding to glutathione (GSH). Initially, exogenous compounds are bound to GSH by glutathione‐*S*‐transferase (GST), which is transported out of cells by MRP1 [15]. Intracellular GSH homeostasis depends not only on its synthesis rate but also on its efflux through plasma membrane transporters [16]. Treatment of MRP1‐expressing cells with verapamil or its derivatives rapidly reduces the intracellular GSH content and promotes cell death [17]. Conversely, the addition of GSH to the medium effectively prevents cell death. Therefore, verapamil and its derivatives induce apoptosis by stimulating MRP1‐mediated GSH excretion [17]. Reduced GSH plays a crucial role in protecting the intracellular environment by protecting cellular macromolecules, such as DNA, proteins, and lipids, against oxidative stressors from environmental factors and cytotoxic agents [18]. GSH depletion increased the sensitivity of cells to ferroptosis‐inducing agents [19].

Ferroptosis is a type of iron‐dependent regulated cell death triggered by excessive lipid peroxidation [20]. Iron‐mediated oxidative damage and subsequent cell membrane impairment are the main causes of ferroptosis, which involves increased iron accumulation, free radical production, fatty acid supply, and lipid peroxidation [21]. The regulation of ferroptosis is based on pathways related to iron metabolism, System Xc^−^/glutathione peroxidase 4 (GPX4), and lipid metabolism [22]. In the System Xc^−^/GPX4 pathway, System Xc^−^ serves as an essential transporter involved in GSH synthesis [23] and GPX4 acts as an antioxidant by catalyzing the conversion of GSH into oxidized glutathione (GSSG), with GSH at the core of this pathway [23]. As a crucial endogenous antioxidant, GSH eliminates reactive oxygen species (ROS) and active nitrogen while functioning as an antidote for complete detoxification purposes [24]. When intracellular levels of GSH fall below a critical threshold, the functionality of the GSH‐dependent glutathione peroxidase 4 (GPX4) is compromised, leading to the potentially fatal accumulation of ROS and subsequent cell death through an iron‐dependent, nonapoptotic process [20, 25]. Ferroptosis is closely related to the pathogenesis of liver fibrosis, and erastin attenuates liver fibrosis by inducing HSCs ferroptosis [26]. Inducing HSC ferroptosis may be a promising therapeutic approach for the treatment and prevention of liver fibrosis. However, selectively inducing HSC ferroptosis without affecting healthy hepatocytes poses a therapeutic challenge. This study aimed at investigating the potential therapeutic roles of AHR and HSC ferroptosis in liver fibrosis.

## 2 Methods

### 2.1 Animal Studies

Wild‐type (WT) C57BL6/J for this study were purchased from Jinan Pengyue Experimental Animal Breeding Co. Ltd. (Jinan, China). Mrp1^fl/fl^ mice (C57BL/6Smoc‐Abcc1em1 (flox) Smoc, stock number NM‐CKO‐233982) were purchased from Shanghai Model Organisms Center Inc. (Shanghai, China) and bred in house. Lrat‐Cre mice (B6.Cg‐Tg(Lrat‐cre)1Rshw/Mmjax, stock number 069595‐JAX) were purchased from the Jackson Laboratory and bred in house. Mrp1^fl/fl^ mice were crossed with Lrat‐Cre mice to obtain mHSC‐specific Mrp1‐deficient mice (Mrp1^−/−^ mice). In CCl_4_‐induced injury experiments, CCl_4_ was diluted with paraffin oil (Aladdin, C116023) (at 1:4 of CCl4 to paraffin oil), and diluted CCl_4_ was injected into mice at a dose of 2 mL/kg body weight. WT mice, Mrp1^fl/fl^ mice or Mrp1^−/−^ mice (males, 6–8 weeks old, *n* = 5 per group), were treated with intraperitoneal paraffin oil injection (control group) or CCl_4_ injection twice per week for 4 weeks and these mice were orally gavaged with either Vehicle (Corn Oil, 0.1 mL/10 g) or YH439 (MCE, HY‐100242, 100 mg/kg/day) once a day for a week. The mice were euthanized under anesthesia, and the liver tissue was cryopreserved using liquid nitrogen or fixed in 10% formalin buffer solution, followed by embedding in paraffin or OCT compound. Additionally, serum samples were collected. All experimental procedures were conducted in accordance with the Laboratory Animal Care and Use Guidelines of Jining Medical College. The entire study adhered to the principles of Replacement, Reduction, and Refinement (3Rs) for animal experiments and was performed under the guidance of respecting the ARRIVE criteria.

### 2.2 Isolation of Primary Mouse Hepatocytes

Wild mice were used to isolate primary hepatocytes, the separation procedure described in our previous study [27].

### 2.3 RNA Isolation and QRT‐PCR

Treat primary mouse hepatocytes or mHSCs, respectively, with DMSO (Veh), YH439 (5 μM), TCDD (wellington, DD‐2378‐S, 5 nM) or CH‐223191 (MCE, HY‐12684, 2 μM) for 24 h. Extract RNA from the liver using the RNA Isolation Kit (Beyotime, Cat# R0017S). Use BeyoRT III First Strand cDNA Synthesis Kit (Beyotime, Cat# D7180L) to synthesize cDNA. The MonAmpTM SYBRGreen qPCR Mix (MQ10201S, Low ROX) is utilized for real‐time PCR analysis, which was conducted using the QuantStudio 3 Real‐Time PCR Instrument (Applied Biosystems, USA). The comparative‐Ct method (2^−△△Ct^ method) was used to calculate the relative level. All sequences of primers are listed in Table S1.

### 2.4 Serum Transaminase Levels and Histological Analysis

Sample processing and staining procedures were described in our previous study [27]. Plasma ALT and AST were analyzed with a GPT/ALT kit (Nanjing Jiancheng, Cat#C009‐2) and a GOT/AST kit (Nanjing Jiancheng, C010‐2), respectively. H&E staining kit (Beyotime, Cat#C0105S) was used for H&E staining.

### 2.5 Immunofluorescent Analysis

The experimental procedure was described in our previous study [27]. For immunohistochemical staining, anti‐αSMA (1:100 dilutions, Proteintech, Cat#14395‐1‐AP RRID AB_2223009) was used as the primary antibody. DyLight 594 Conjugated AffiniPure Goat Anti‐rabbit IgG (H + L) (1:500 dilution) were used as secondary antibodies. DAPI (Abcam, Cat# ab104139) staining the nucleus. A laser scanning confocal microscope (LSM710, Carl Zeiss microscope) was used to capture the images.

### 2.6 Software–Immunofluorescence Staining Quantification

Image Pro Plus (Image Pro Plus v.7: Media Cybernetics; Bethesda, MD), is an analytical program used to analyze and quantify data in micrographs.

### 2.7 EMSA

The experimental procedure was described in our previous study [27]. mHSCs were treated with YH439 (5 μM) for 24 h. The Nuclear and Cytoplasmic Protein Extraction Kit (Beyotime, P0027) is used to extract nuclear proteins. The electrophoretic mobility shift assay (EMSA) Kit (Beyotime GS009) is used to detect the DNA binding activity of AHR. Single‐stranded oligonucleotides were synthesized by Sangon. The sequences are listed in Table S1.

### 2.8 Western Blot

The experimental procedure was described in our previous study [27]. Primary antibody: AHR (1:2000 dilution, BOSTER Cat# A00225‐4, RRID: AB_3095576). MRP1 (1:2000 dilution, Proteintech, Cat# 67228‐1‐Ig, RRID: AB_2882516), GAPDH (1:2000 dilution, Proteintech, Cat# 10494‐1‐AP, RRID: AB_2263076), CYP1A1 (1:2000 dilution Proteintech, Cat# 13241‐1‐AP, RRID: AB_2877928). Secondary antibodies: HRP‐conjugated Affinipure Goat Anti‐Mouse IgG(H + L) (1:2000 dilution, Proteintech, Cat# SA00001‐1, RRID: AB_2722565). HRP‐conjugated Affinipure Goat Anti‐Rabbit IgG(H + L) (1:2000 dilution, Proteintech, Cat# SA00001‐2, RRID: AB_2722564).

### 2.9 ChIP

The experimental procedure was described in our previous study [27]. ChIP assays were performed using the ChIP Assay kit (Beyotime, P2078). mHSCs were treated with YH439 (5 μM) for 24 h. Subsequently, mHSCs were sonicated and then immunoprecipitated with the antibody against AHR (1:100 dilution BOSTER Cat# A00225‐4, RRID: AB_3095576) with IgG (1:100 dilution Proteintech Cat# 30000‐0‐AP RRID AB_2819035) as a negative control. The primer sequences used for PCR amplification are listed in Table S1.

### 2.10 Molecular Cloning and Cell‐Based Luciferase Reporter Assay

Amplify and insert the Mrp1 promoter fragment into the pGL3‐basic vector (Promega, E1751). Perform site‐directed mutagenesis on the recombinant plasmid. The above plasmids together with the phRL‐TK (Promega, E2241) were co‐transfected into Hep1‐6 cells, using Lipofectamine 2000 (Invitrogen, 11668019). After 6 h incubation, the cells were treated with DMSO (Veh), YH439 (1 μM) for 24 h. Use the dual‐luciferase assay kit (Promega, E1910) to measure luciferase activity. Measure the enzyme activity of luciferase using a Fluoroskan Ascent FL (Thermo Scientific, USA). The primer sequences are listed in Table S1.

### 2.11 Electron Microscopy

Primary mouse hepatocytes or mHSCs, respectively, were treated with DMSO (Veh), YH439 (5 μM) for 24 h to transmission electron microscopic. Sample processing and staining procedures were described in our previous study [27]. Images were obtained using transmission electron microscopy (FEI, TECNAI G2 20 TWIN, USA).

### 2.12 Cell Culture and miRNA Transfection

The cell lines used in this study included primary mouse hepatocytes, Hep1‐6 cells (ATCC, CRL‐1830), or mHSCs (ScienCell Research Laboratories, M5300‐57). Primary mouse hepatocytes and Hep1‐6 were grown in high glucose DMEM (Hyclone, SH30243.01B) or DMEM Phenol Red‐Free (Hyclone, SH30284.02). mHSCs were grown in RPMI 1640 (Hyclone, SH30096.01) or RPMI 1640 Phenol Red‐Free (Hyclone, SH30605.01) supplied with 10% (vol/vol) fetal bovine serum and 1% (vol/vol) penicillin–streptomycin. miRNAs were transfected at a concentration of 30 nM using Lipofectamine RNAiMAX (Invitrogen, Carlsbad, CA, USA) according to the manufacturer's instructions. The siRNA‐ctrl, siRNA‐Mrp1, or siRNA‐Ahr were purchased from Gene Pharma (Shanghai, China). The primer sequences are listed in Table S1.

### 2.13 GSH Detection

Primary mouse hepatocytes or mHSCs were grown in DMEM Phenol Red‐Free (Hyclone, SH30284.02) or RPMI 1640 Phenol Red‐Free (Hyclone, SH30605.01). Primary mouse hepatocytes or mHSCs, respectively, were treated with DMSO (Veh), YH439 (5 μM) or H_2_O (Veh), and ATP (2 mM) for 24 h. The levels of Intracellular GSH were measured by Reduced Glutathione (GSH) Content Assay Kit (Solarbio BC1175) following the manufacturer's instruction. The cell medium was collected and centrifuged at room temperature at 12,000 g for 10 min. The supernatant was collected for the detection of extracellular GSH levels. The levels of extracellular GSH were measured by Reduced Glutathione (GSH) Content Assay Kit (Solarbio BC1175) following the manufacturer's instruction.

### 2.14 GPXs Activity Assay Using Tert‐Butylhydroperoxide

Primary mouse hepatocytes or mHSCs, respectively, were treated with DMSO (Veh) and YH439 (5 μM) for 24 h to measure the activity of GPXs. The experimental procedure was described in the previous study [25].

### 2.15 ROS, MDA, Fe^2+^, Cell Death, and MMP Detection

Primary mouse hepatocytes or mHSCs, respectively, treated with DMSO (Veh) and YH439 (5 μM) for 24 h. Discard the supernatant and wash the cells three times with PBS. H2DCFDA (2 μm, MCE, Cat# HY‐D0940), BODIPY 581/591 C11 (2 μm, MCE, HY‐D1301), FerroOrange (1 μm, MKBio, Cat#MX4559), or SYTOX Green (1 μm, Thermo Fisher Scientific, Cat#S7020) were incubated for 30 min, respectively. DAPI (Abcam, Cat# ab104139) was used for staining the nucleus. The levels of MMP were measured by Mitochondrial Membrane Potential Assay Kit with TMRE (Beyotime, C2001S) following the manufacturer's instruction. A laser scanning confocal microscope (LSM710, Carl Zeiss microscope) was used to capture the images.

### 2.16 Quantitative and Statistical Analyses

*Statistical analysis*: Statistical analyses were performed using the GraphPad Prism 6 (GraphPad). Data are expressed as means ± SD. Comparisons between the two groups were performed using two‐tailed Student's *t*‐tests. Comparisons between multiple groups were performed using ordinary one‐way ANOVA with Dunnett's multiple comparison tests. Statistical significance was presented at the level of *p < 0.05, **p < 0.01, ***p < 0.001.

## 3 Results

### 3.1 *Mrp1* Is a Target Gene of AHR

**FIGURE 1:**
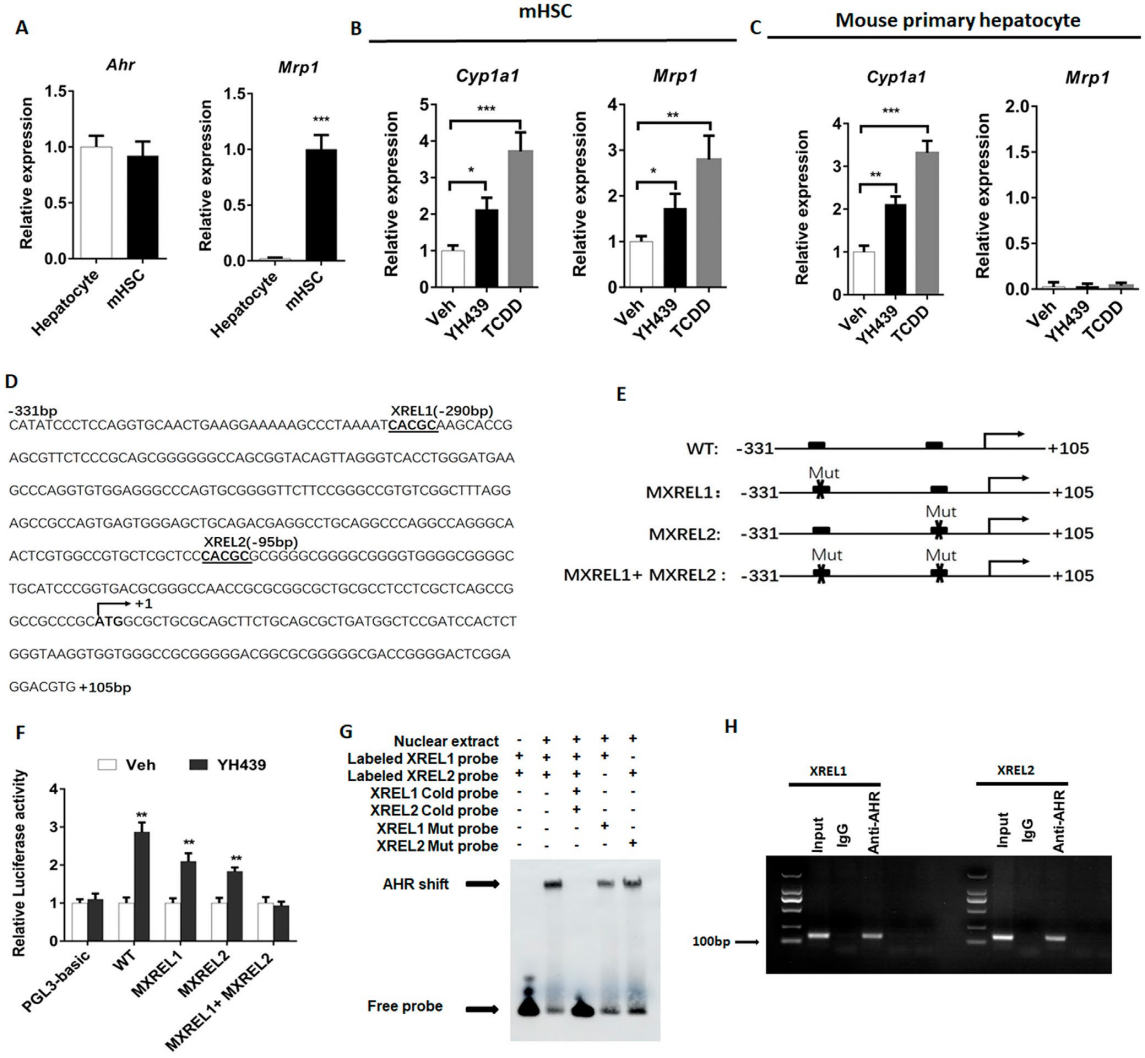
AHR directly regulates Mrp1 transcription in mHSCs. (A–C) The expression of Ahr, Mrp1 or Cyp1a1 was detected by QPCR. (D) There are two potential AHR exogenous response elements on the Mrp1 promoter sequence. (E) Promoter sequences containing XREL1 and XREL2 were cloned onto PGL3 plasmids, and mutant plasmids were constructed. (F) Double luciferase assay to detect luciferase activity. (G) EMSA detects AHR binding to specific elements XREL1 and XREL2. (H) CHIP detects AHR binding to specific elements XREL1 and XREL2. Data are expressed as means ± SD; *p < 0.05, **p < 0.01 and ***p < 0.001; Student's *t*‐test or one‐way ANOVA.

First, we measured the expression of *Ahr* and *Mrp1* in primary mouse hepatocytes and mHSCs. *Ahr* was expressed in mHSCs and primary mouse hepatocytes (Figure 1A and Figure S1A). *Mrp1* was expressed in mHSCs, but not in primary mouse hepatocytes (Figure 1A and Figure S1A). To confirm that AHR regulates the expression of *Mrp1*, we treated mHSCs and primary mouse hepatocytes with AHR agonists, YH439 or TCDD. Cytochrome P4501A1 (*Cyp1a1*) is a target of AHR. We found that *Cyp1a1* expression was upregulated after YH439 or TCDD treatment in both mHSCs and primary mouse hepatocytes, indicating the activation of AHR (Figure 1B,C and Figure S1B). *Mrp1* expression was upregulated after the treatment of mHSCs with YH439 or TCDD (Figure 1B and Figure S1B). Compared with YH439 treatment, the expression of *Cyp1a1* and *Mrp1* were slightly higher after TCDD treatment (Figure 1B). However, the expression of *Mrp1* was not detected in mouse primary hepatocytes. Furthermore, the treatment with YH439 did not induce the expression of *Mrp1* in mouse primary hepatocytes (Figure 1C). Two potential exogenous reaction elements for AHR were identified in the promoter sequence of *Mrp1*, with a core sequence of 5′‐GCGTG‐3′ (Figure 1D). The promoter region containing XREL1 and XREL2 binding sites (−331 to +105 bp) was cloned into the PGL3 plasmid, and a mutant plasmid was constructed simultaneously (Figure 1E). Dual‐luciferase experiments showed that the presence of both XREL1 and XREL2 binding sites increased luciferase activity after YH439 treatment, but there was no increase in luciferase activity after mutation of these two binding sites (Figure 1F). These results indicate that AHR directly regulates *Mrp1* transcription by binding to these response elements within the promoter region. To further elucidate the specific elements XREL1 and XREL2 in the *Mrp1* promoter as AHR response elements, we treated mHSCs with YH439 and conducted electrophoretic mobility shift assays (EMSA). The EMSA results revealed that labeled XREL1 and XREL2 probes interacted with the nuclear extracts of YH439‐treated mHSCs, resulting in the expected DNA/protein migration bands. The intensity of the DNA/protein migration band was significantly attenuated upon the addition of the unlabeled (cold) probe, whereas no change was observed when the mutant probe was added (Figure 1G). These findings indicate that XREL1 and XREL2 in the *Mrp1* promoter function as AHR‐responsive elements. Chromatin immunoprecipitation (ChIP) experiments were performed to further confirm binding between AHR and XREL1/XREL2 within the *Mrp1* promoter region. In YH439‐treated mHSCs, the amplification of DNA fragments containing XREL1 and XREL2 was observed in the anti‐AHR group (Figure 1H), confirming the direct regulatory influence of AHR on *Mrp1* gene expression. Silencing *Ahr* expression abolished the effects of YH439 on *Mrp1* expression in mHSCs (Figure S1C,D). Furthermore, treatment with the AHR antagonist CH‐223191 mitigated the effects of YH439 on *Mrp1* expression in mHSCs (Figure S1E,F). These results indicate that AHR directly regulates the expression of *Mrp1*.

### 3.2 AHR Promotes GSH Efflux

**FIGURE 2:**
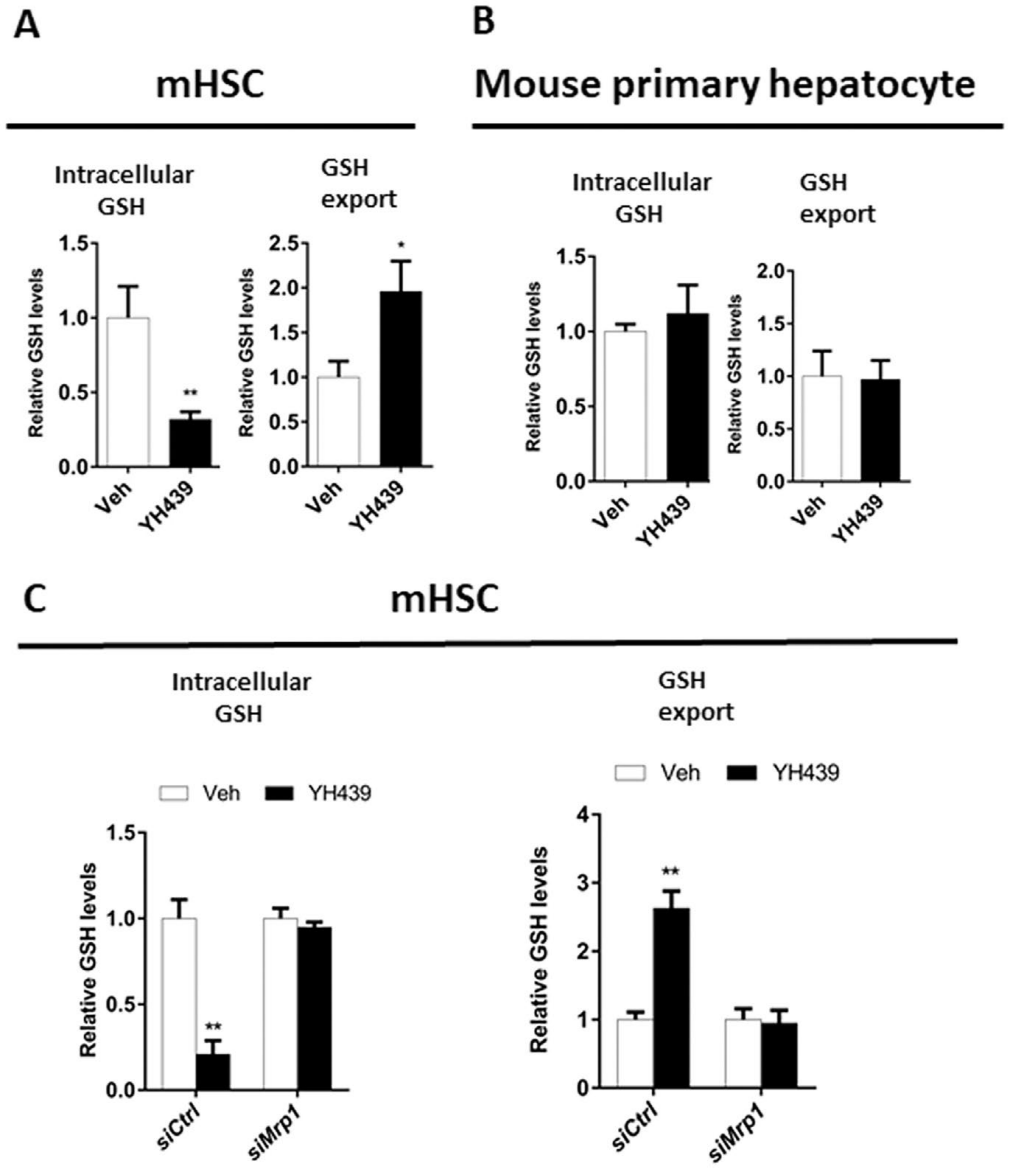
AHR reduces the antioxidant capacity of mHSCs. (A) Analysis of GSH content changes. (B) Analysis of GSH content changes. (C) Analysis of GSH content changes. Data are expressed as means ± SD; *p < 0.05 and **p < 0.01; Student's *t*‐test.

YH439 treatment decreased intracellular GSH content and induced GSH expulsion in mHSCs, leading to an increase in extracellular GSH content (Figure 2A). However, AHR activation had no effect on intracellular or extracellular GSH content in primary mouse hepatocytes (Figure 2B). Silencing *Mrp1* expression abolished the effects of AHR on GSH transport (Figure 2C), indicating that AHR promotes GSH efflux by regulating *Mrp1* expression.

mHSCs and mouse primary hepatocytes were treated with YH439, and the results revealed a significant upregulation of *Cpt1a* and *Acox1* expression (Figure S2A,D). These findings suggest that AHR may enhance mitochondrial oxidative phosphorylation. Subsequently, the ATP content of YH439‐treated mHSCs and primary mouse hepatocytes was determined, and the results showed that AHR enhanced ATP production (Figure S2B,E). As *Mrp1*‐mediated transport relies on ATP, these results indicate that AHR facilitates GSH efflux by promoting mitochondrial oxidative phosphorylation to generate sufficient levels of ATP. To further validate the role of ATP in facilitating GSH efflux via *Mrp1*, mHSCs and mouse primary hepatocytes were treated with exogenous ATP. Notably, exogenous ATP treatment led to a decrease in intracellular GSH content in mHSCs and an increase in extracellular GSH content (Figure S2C), whereas no significant changes were observed in intracellular or extracellular GSH content in mouse primary hepatocytes upon exogenous ATP treatment (Figure S2F). Collectively, these results demonstrate that ATP promotes *Mrp1*‐mediated GSH efflux.

### 3.3 AHR Induces mHSC Ferroptosis

**FIGURE 3:**
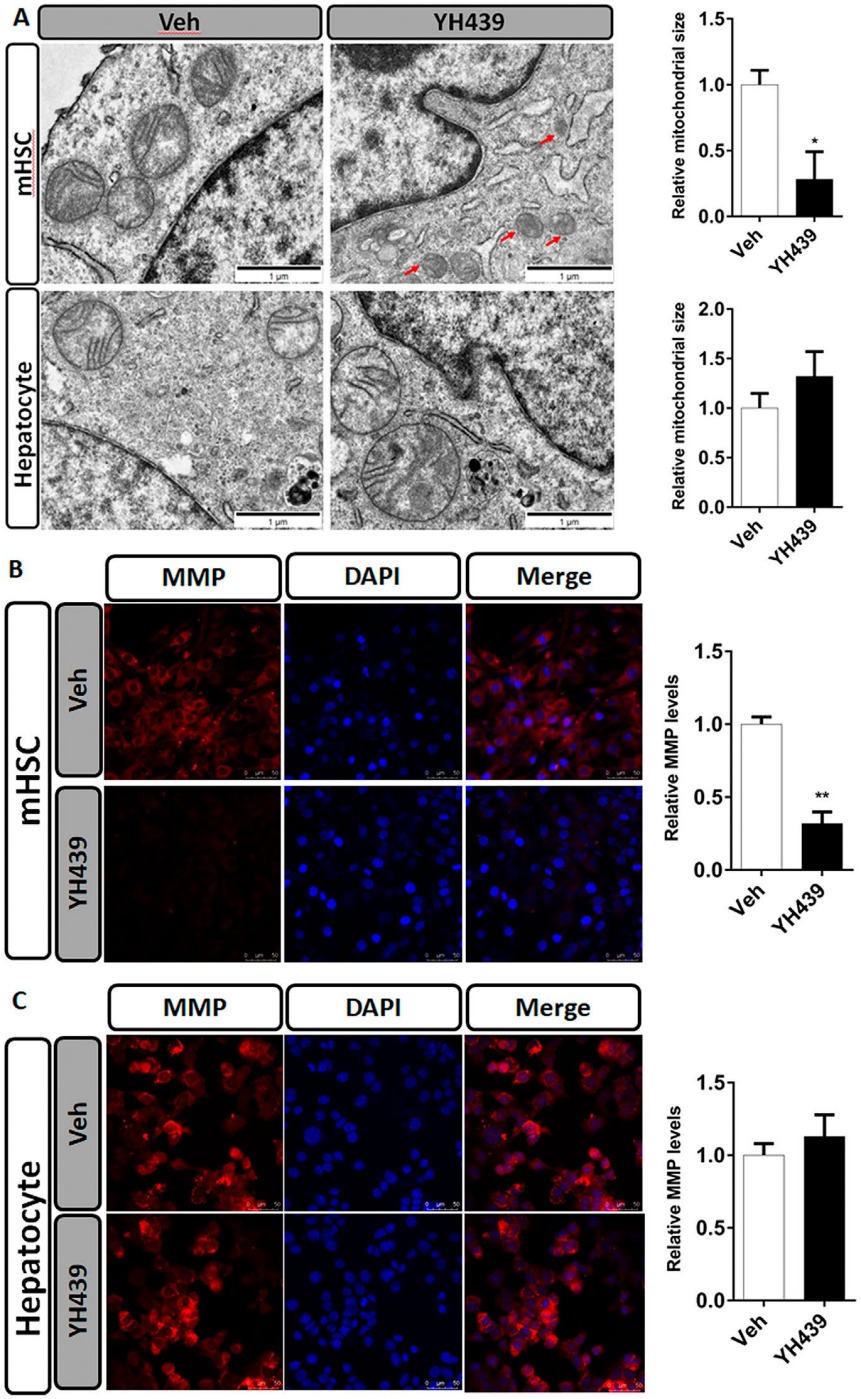
AHR increases the sensitivity of HSC to ferroptosis. (A) Morphological changes in mitochondria were detected by electron microscopy. Quantification of mitochondria sizes. Scale bar = 1 μm. (B) TMRE fluorescent probe was used to analyze the change of mitochondrial membrane potential in mHSCs. The change of mitochondrial membrane potential in mHSCs was quantitatively analyzed. Scale bar = 50 μm (C) TMRE fluorescent probe was used to analyze the change of mitochondrial membrane potential in primary mouse hepatocytes. The change of mitochondrial membrane potential in primary mouse hepatocytes was quantitatively analyzed. Data are expressed as means ± SD; *p < 0.05 and **p < 0.01; Student's *t*‐test. Scale bar = 50 μm.

mHSCs and mouse primary hepatocytes were treated with YH439, and changes in mitochondrial morphology and mitochondrial membrane potential were examined. AHR activation led to a reduction in mitochondrial size, the disappearance of cristae, and a decrease in membrane potential (Figure 3A,B), which may ultimately promot ferroptosis specifically in mHSCs. However, no significant changes were observed in the mitochondrial morphology or membrane potential of mouse primary hepatocytes (Figure 3A,C).

**FIGURE 4:**
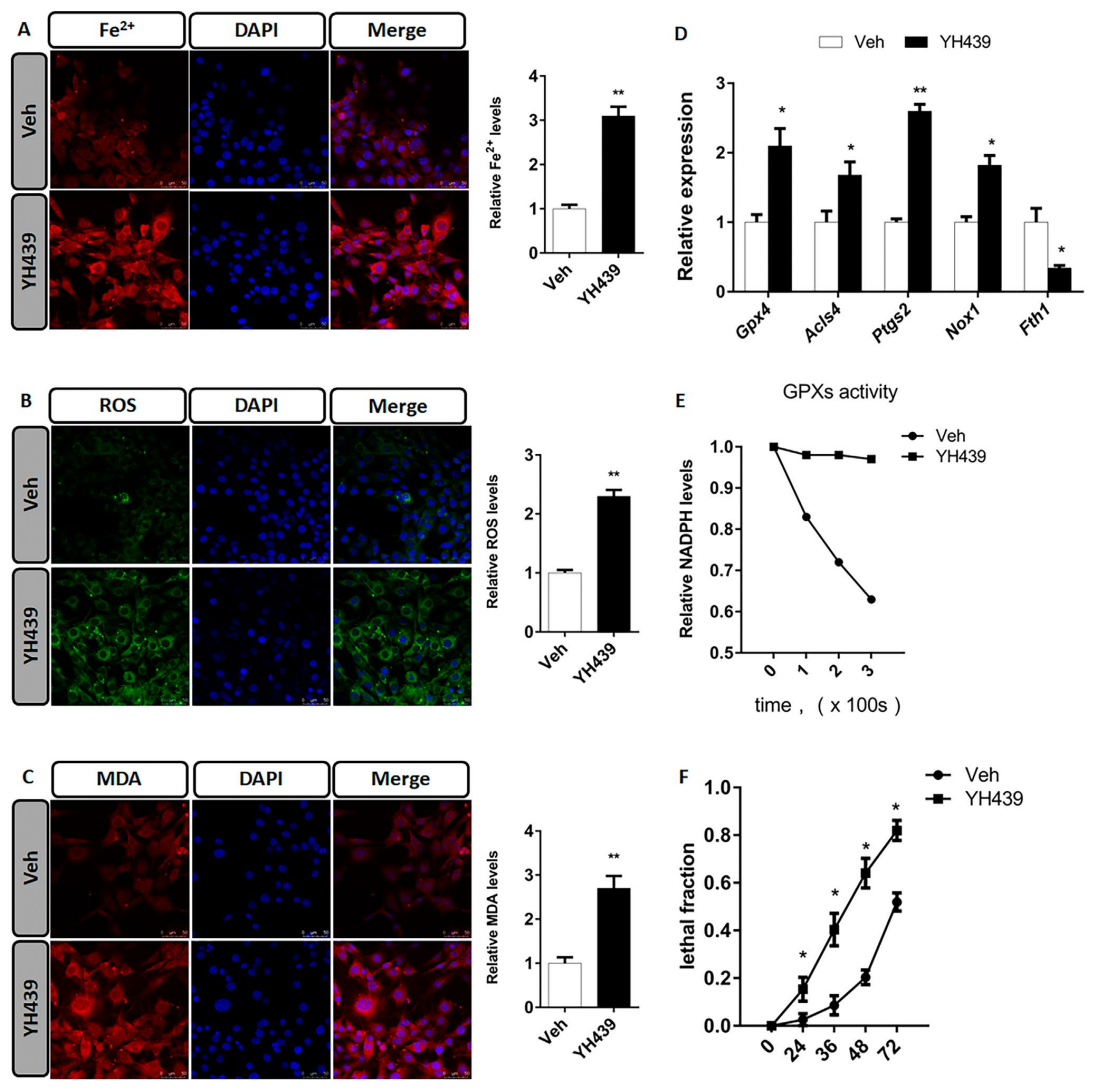
AHR promotes mHSC ferroptosis. (A) Ferroorange fluorescent probe was used to analyze the change of Fe^2+^ content in mHSCs. The change of Fe^2+^ content in HSCs was quantitatively analyzed. (B) H2DCFDA fluorescent probe was used to analyze the change of ROS content in mHSCs. The change of ROS content in HSCs was quantitatively analyzed. (C) BODIPY 581/591 C11 fluorescent probe was used to analyze the change of MDA content in mHSCs. The change of MDA content in mHSCs was quantitatively analyzed. (D) QPCR analysis of iron death related gene expression changes. (E) Analysis of intracellular GPXs activity. (F) Analysis of cell mortality. Data are expressed as means ± SD; *p < 0.05 and **p < 0.01; Student's *t*‐test. Scale bar = 50 μm.

Next, we found that treatment of mHSCs with YH439 increased the levels of Fe^2+^, ROS, and malondialdehyde (MDA) (Figure 4A–C). To demonstrate the role of AHR in promoting ferroptosis of mHSCs, we treated mHSCs with YH439 and examined the expression of ferroptosis‐related genes, including *Gpx4*, *Acsl4*, *Ptgs2*, *Nox1*, and *Fth1*. We observed an upregulation of *Gpx4 Acsl4, Ptgs2*, and *Nox1* in mHSCs (Figure 4D). Additionally, *Fth1* expression was downregulated in mHSCs (Figure 4D). Upregulation of Gpx4 appeared to interfere with ferroptosis in mHSCs. Previous studies have reported that GPX4 activity depend on intracellular GSH levels above a certain threshold [25]. After YH439 treatment of mHSCs, we observed an increase in *Mrp1* expression which promotes GSH efflux. These findings suggest that although *Gpx4* expression is upregulated, *Mrp1*‐mediated GSH efflux leads to decreased intracellular GSH content, which may limit GPX4 activity and promote ferroptosis in mHSCs. We validated the hypothesis that GSH efflux leads to the depletion of intracellular GSH content, thereby impairing GPX4 activity. Initially, *siMrp1* was ued to silence *Mrp1* expression in mHSCs and was followed by YH439 treatment to assess intracellular ROS levels. These findings revealed that activation of AHR in the control group led to an increase in intracellular ROS levels. However, after silencing *Mrp1*, AHR activation led to a decrease in intracellular ROS content (Figure S3). Thus, AHR promotes the expression of *Mrp1* and enhances GSH efflux, while reducing its cellular content, thus inhibiting GPX4 activity and increasing ROS levels. Conversely, silencing *Mrp1* impeded GSH efflux, increased intracellular GSH levels, promoted GPX4 activity, and subsequently reduces ROS content. To further confirm that AHR facilitates GSH efflux through upregulation of *Mrp1* expression while suppressing GPX4 activity by decreasing GSH levels. mHSCs were treated with YH439, and the total activity of GPXs in mHSCs was detected using tert‐butylhydrogen peroxide (tBuOOH) as a substrate, by monitoring the NADPH oxidation rate, which was coupled with the tBuOOH reducing activity of GPXs in the cell lysate. When GPXs activity was measured in the mHSC lysate of the control group, we observed a decrease in NADPH content, indicating that GPXs reduced tBuOOH in the lysate and exhibited activity. However, when GPXs activity was measured in the YH439‐treated mHSC lysate, NADPH oxidation was prevented, indicating that GPXs were inactivated upon GSH depletion (Figure 4E). Based on these findings, it can be concluded that AHR reduces intracellular GSH content by promoting *Mrp1* expression and enhancing GSH efflux, thus inhibiting GPX4 activity. Therefore, the upregulation of *Gpx4* is not contradictory to mHSC ferroptosis. Furthermore, we examined the effects of YH439 on mHSC mortality and found that YH439 promoted mHSC death (Figure 4F). Based on these results, it can be preliminarily inferred that YH439 promotes the efflux of GSH by activating AHR to the expression of *Mrp1*, leading to a decreased antioxidant capacity of mHSC and facilitating ferroptosis.

**FIGURE 5:**
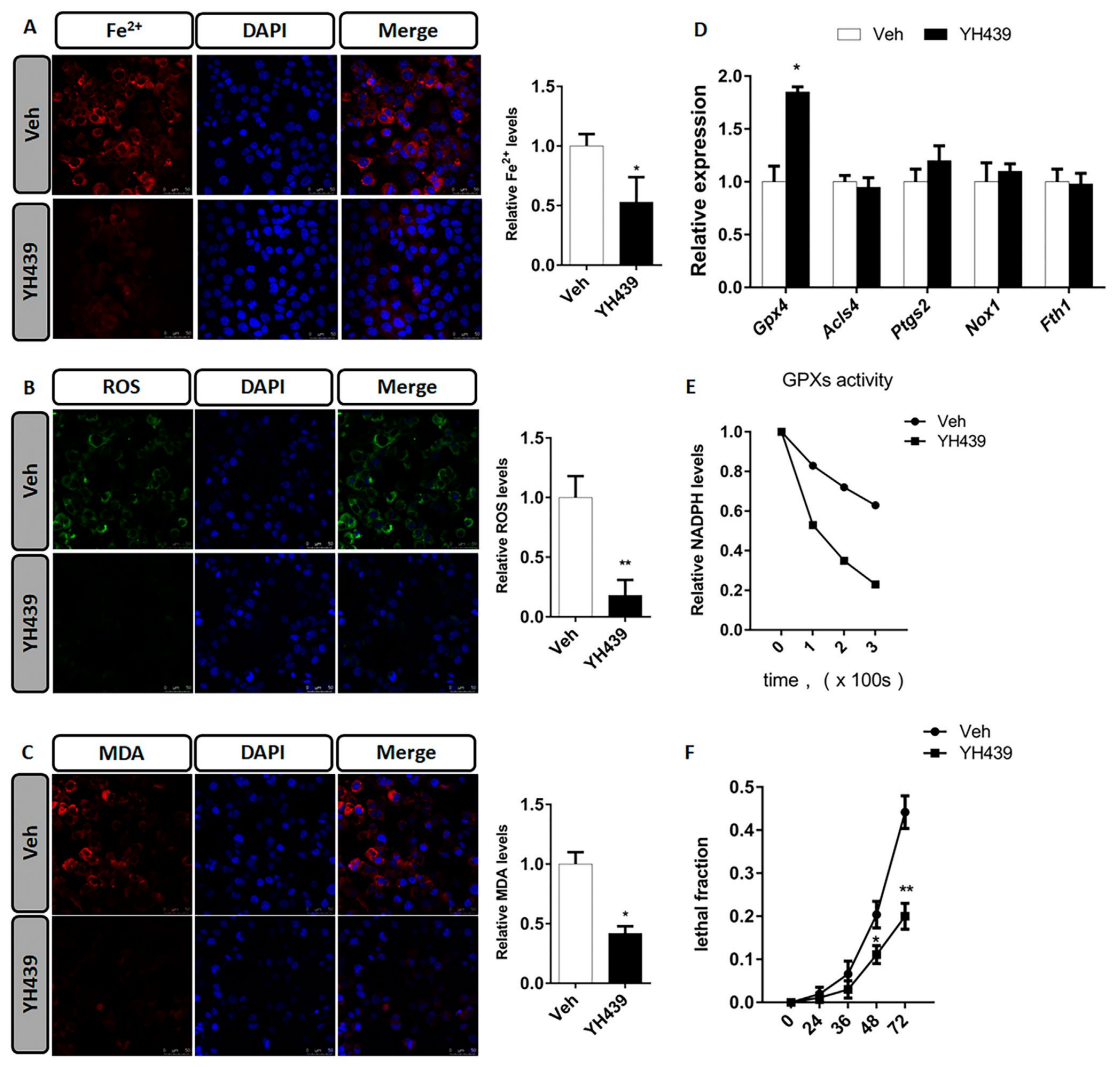
AHR improved the antioxidant capacity of primary mouse hepatocytes. (A) Ferroorange fluorescent probe was used to analyze the change of Fe^2+^ content in primary mouse hepatocytes. The change of Fe^2+^ content in primary mouse hepatocytes was quantitatively analyzed. (B) H2DCFDA fluorescent probe was used to analyze the change of ROS content in primary mouse hepatocytes. The change of ROS content in primary mouse hepatocytes was quantitatively analyzed. (C) BODIPY 581/591 C11 fluorescent probe was used to analyze the change of MDA content in primary mouse hepatocytes. The change of MDA content in primary mouse hepatocytes was quantitatively analyzed. (D) QPCR analysis of iron death related gene expression changes in primary mouse hepatocytes. (E) Analysis of intracellular GPXs activity. (F) Analysis of cell mortality. Data are expressed as means ± SD; *p < 0.05 and **p < 0.01; Student's *t*‐test. Scale bar = 50 μm.

YH439 treatment decreased the levels of Fe^2+^, ROS, and MDA within primary mouse hepatocytes (Figure 5A–C). Primary mouse hepatocytes were treated with YH439 to detect the expression of ferroptosis‐related genes: *Gpx4, Acsl4, Ptgs2, Nox1*, and *Fth1*. Notably, *Gpx4* expression was upregulated in mouse primary hepatocytes. In contrast, there were no significant changes in the expression levels of *Acsl4*, *Ptgs2*, *Nox1*, and *Fth1* (Figure 5D). Finally, we assessed the overall activity of GPXs in mouse primary hepatocytes after treatment with YH439. When GPXs activity was measured in the primary mouse hepatocyte lysate of the control group, we observed a decrease in NADPH content, indicating that GPXs reduced tBuOOH in the cell lysate. When GPXs activity was measured in YH439‐treated mouse primary hepatocyte lysates, we observed lower NADPH content compared to that in the control group, suggesting that high levels of GPXs in the lysate facilitated faster reduction of tBuOOH (Figure 5E). Based on these results, it can be inferred that AHR increases the antioxidant capacity of primary mouse hepatocytes by upregulating the GPX4 expression. Treatment with YH439 also reduced the mortality of primary mouse hepatocytes (Figure 5F). Collectively, these findings preliminarily confirm that YH439 activates AHR to promote GXP4 expression, thus increasing antioxidant capacity and inhibiting ferroptosis in primary mouse hepatocytes.

### 3.4 AHR Alleviates Liver Fibrosis in Mice

**FIGURE 6:**
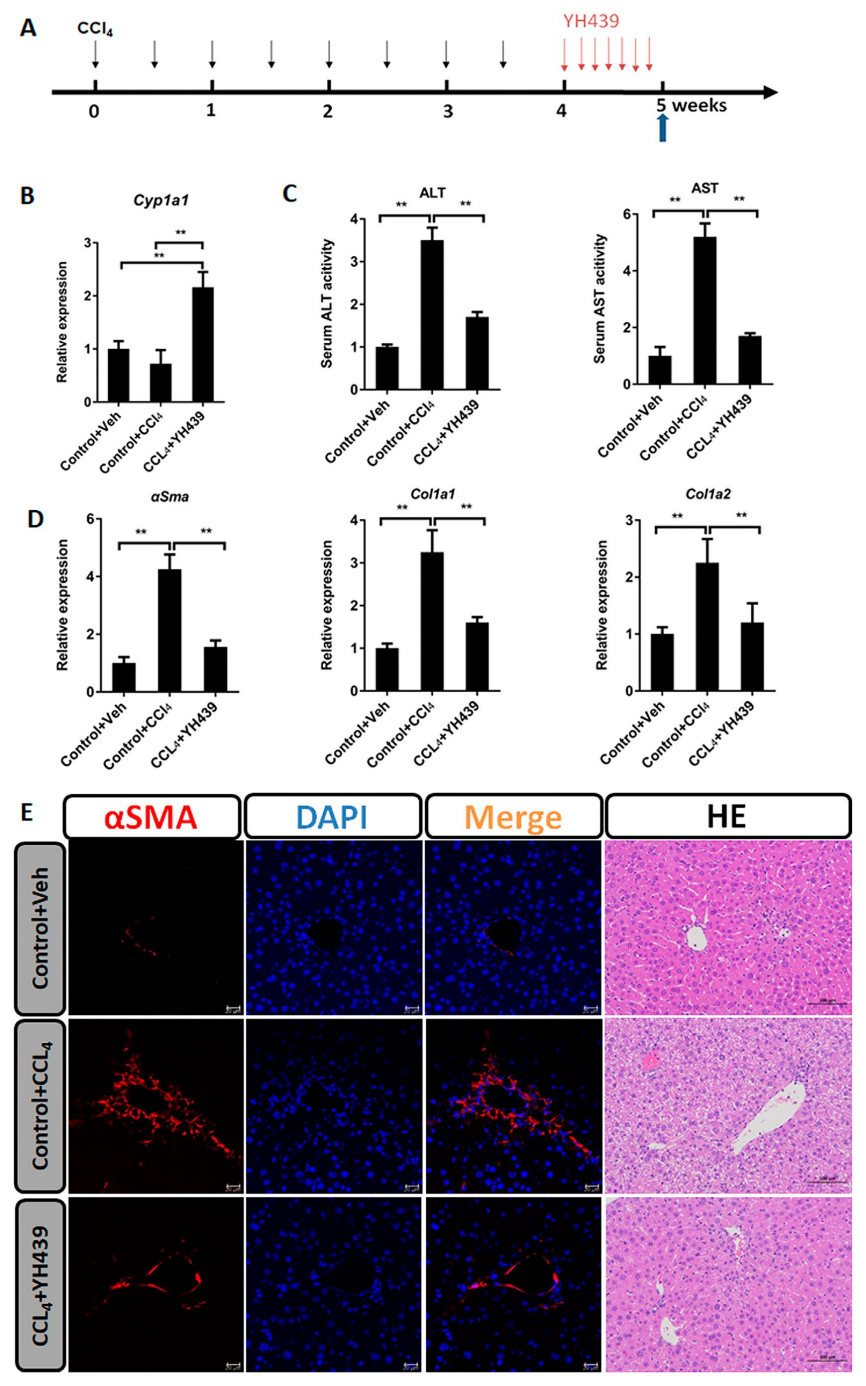
AHR alleviates liver fibrosis. (A) The WT mice received intraperitoneal paraffin oil injection (control group) or CCl4 injection twice per week for 4 weeks and these mice were orally gavaged with either Vehicle or YH439 once a day for a week. (B) Expressions of *Cyp1a1* were detected by QPCR (*n* = 5). (C) The changes in ALT and AST levels were detected (*n* = 5). (D) Expressions of *αSma, Col1a1*, and *Col1a2* were detected by QPCR (*n* = 5). (E) αSMA Immunofluorescence staining and H&E staining. The scales are 50 or 100 μm, respectively. Data are expressed as means ± SD; *p < 0.05 and **p < 0.01; one‐way ANOVA.

A mouse model of chronic liver fibrosis was established using wild type (WT) mice. CCL_4_ was administered intraperitoneally twice a week for 4 weeks, followed by oral administration of YH439 once a day for 1 week (Figure 6A). *Cyp1a1* expression was upregulated after YH439 treatment (Figure 6B). YH439 reduced AST and ALT levels (Figure 6C), indicating its ability to alleviate liver injury. YH439 inhibited the expression of *αSma, Col1a1*, and *Col1a2* (Figure 6D). αSMA staining showed an increase in αSMA expression in the CCL_4_ group, However, αSMA expression decreased in the CCL_4_ + YH439 group. Hematoxylin and eosin (H&E) staining revealed that YH439 reduced liver damage (Figure 6E). Sirius Red staining also showed that YH439 alleviated CCl_4_‐induced liver fibrosis (Figure S4A). Furthermore, we also found that YH439 can reduce the expression of inflammatory genes tumour necrosis factor‐alpha (TNF‐α), interferon‐gamma (IFN‐γ), and interleukin‐6 (IL‐6) in the mouse model of chronic liver fibrosis (Figure S4B). These findings suggest that AHR activation can alleviate liver fibrosis and associated injury.

**FIGURE 7:**
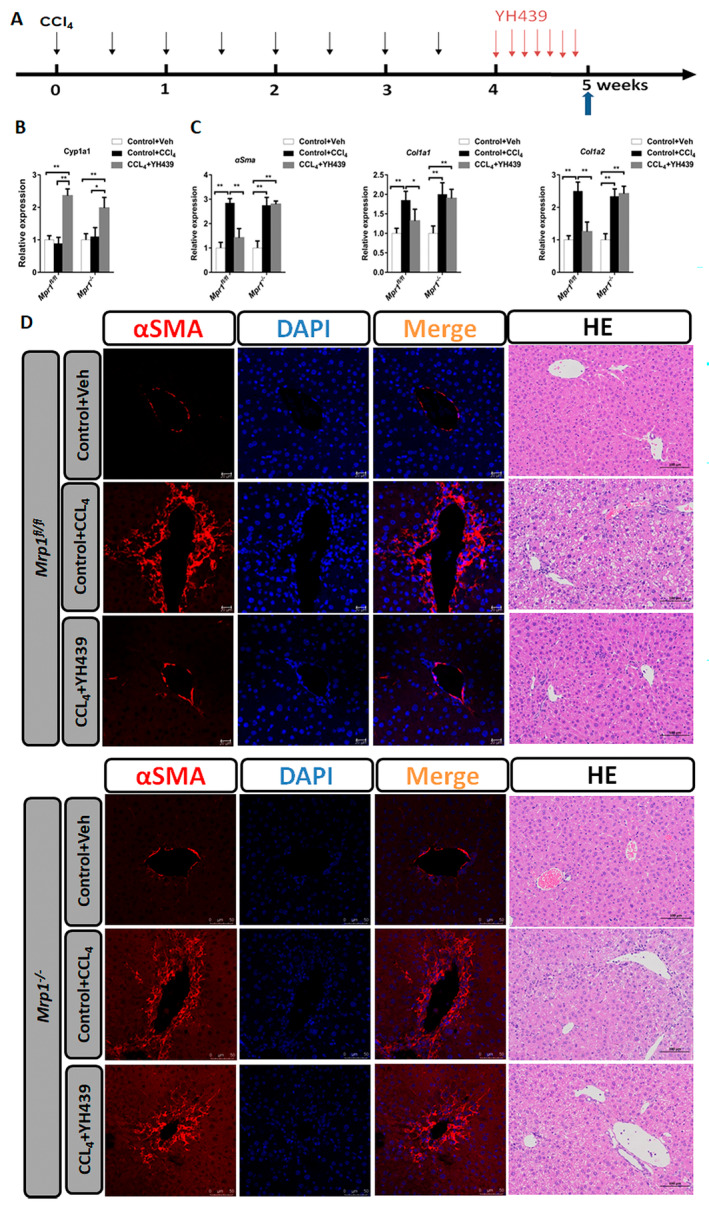
AHR alleviates liver fibrosis and is abolished in HSC‐specific Mrp1 deficient (Mrp1^−/−^) mice. (A)The Mrp1^fl/fl^ mice or Mrp1^−/−^ mice received intraperitoneal paraffin oil injection (control group) or CCl4 injection twice per week for 4 weeks and these mice were orally gavaged with either Vehicle or YH439 once a day for a week. (B) Expressions of *Cyp1a1* were detected by QPCR (*n* = 5). (C) Expressions of *αSma, Col1a1*, and *Col1a2* were detected by QPCR (*n* = 5). (D) αSMA Immunofluorescence staining and H&E staining. The scales are 50 μm or 100 μm, respectively. Data are expressed as means ± SD; *p < 0.05 and **p < 0.01; one‐way ANOVA.

A mouse model of chronic liver fibrosis was created. *Mrp1* ^*fl/fl*^ and *Mrp1* ^−/−^ mice were administered of CCL_4_ intraperitoneally twice a week for 4 weeks, followed by oral administration of YH439 once a day for 1 week (Figure 7A). *Cyp1a1* expression was upregulated after YH439 treatment in *Mrp1* ^*fl/fl*^ mice or *Mrp1* ^−/−^ (Figure 7B). In *Mrp1* ^*fl/fl*^ mice, treatment with YH439 significantly reduced the expression of *αSma*, *Col1a1*, and *Col1a2* (Figure 7C), as well as AST and ALT levels (Figure S5A). No such effects were observed in *Mrp1* ^−/−^ mice (Figure 7C and Figure S5A). In *Mrp1* ^*fl/fl*^ mice, immunofluorescence staining for αSMA demonstrated a significant reduction in αSMA expression after YH439 treatment (Figure 7D). Furthermore, H&E staining revealed that YH439 improved liver damage (Figure 7D). In contrast, such alterations were not observed in *Mrp1* ^−/−^ mice (Figure 7D). In *Mrp1* ^*fl/fl*^ mice, Sirius red staining results also showed that YH439 alleviated CCl_4_‐induced liver fibrosis, but this effect was not observed in *Mrp1* ^−/−^ mice (Figure S5B). Collectively, based on the above in vivo and in vitro experiments, we can conclude that in the chronic liver fibrosis model, the nontoxic ligand YH439 activates AHR to promote ferroptosis of mHSCs, but does not cause ferroptosis in healthy hepatocytes, thus alleviating liver fibrosis.

## 4 Discussion

Chronic liver injury caused by various factors can lead to liver fibrosis [1, 28]. However, the lack of precise therapeutic targets and effective target validation methods poses a significant challenge for the successful treatment of liver fibrosis. Research on liver fibrosis treatment has focused on HSC ferroptosis, HSC apoptosis, HSC senescence, or reversion to a quiescent state [26, 29, 30]. We found that the nontoxic ligand YH439 directly regulated the expression of *Mrp1* in mHSCs by activating AHR, thereby reducing their antioxidant capacity by promoting GSH efflux, and inducing ferroptosis specifically in mHSCs, without causing hepatocyte ferroptosis. In a chronic liver fibrosis model, AHR alleviated liver fibrosis by facilitating ferroptosis in mHSCs without causing hepatocyte ferroptosis. This study provides new information on the role of AHR‐mediated GSH efflux in reducing the antioxidant capacity of mHSCs and promoting mHSC ferroptosis. Furthermore, this study elucidated the molecular mechanism by which AHR alleviates liver fibrosis and provides a potential target for the treatment of liver fibrosis.

The role of AHR in liver fibrosis remains controversial. Spontaneous liver fibrosis can be observed in AHR‐knockout mice [31]. Conversely, the activation of AHR by TCDD can induce liver fibrosis in mice [32]. The nontoxic endogenous ligand ITE inhibits mHSC activation via an AHR‐dependent mechanism. However, the specific mechanisms underlying the different effects of TCDD and ITE on mHSC activation and liver fibrosis remain unclear [8]. This study found that, compared to YH439 treatment, the expression of *Cyp1a1* and *Mrp1* were slightly higher after TCDD treatment. The metabolic stability of TCDD and YH439 is different. TCDD has a stable metabolism and an estimated half‐life of more than 7 years in human serum. It has high affinity for AHR and can continuously activate AHR [33]. YH439 has a short half‐life in rat serum [34]. Sustained activation of AHR by exogenous ligands, such as TCDD, and transient activation by endogenous ligands, such as YH439 or ITE, may explain the differential effects between these ligands. One study found that after YH439 administration, *Cyp1a1* mRNA levels peaked at 8 h and then returned to control levels at 16 h. The level of CYP1A1 protein reached its maximum value 24 h after YH439 treatment and returned to near control levels within 72 h [35]. We administered YH439 orally once a day for 1 week in a mouse model of chronic liver injury. Our results showed that *Cyp1a1* expression increased significantly after YH439 treatment. YH439 activates AHR and alleviates liver fibrosis. This study revealed that YH439 specifically promotes ferroptosis in mHSCs by activating AHR without inducing ferroptosis of hepatocytes, thus alleviating liver fibrosis. Developing nontoxic agonists of AHR and investigating their underlying mechanisms may have positive implications for the prevention or treatment of liver fibrosis.

*Mrp1* is a critical regulator of ferroptosis [16, 36]. Elevated GSH levels inhibit ferroptosis by improving GPX4 activity. *Mrp1* overexpression leads to elevated GSH efflux, which disrupts intracellular GSH homeostasis and inhibits GPX4 activity [16, 37]. Increased expression of Mrp1 makes cancer cells susceptible to various ferroptosis inducers, suggesting that targeting the ferroptosis pathway could be a potential strategy to selectively eliminate cancer cells with elevated Mrp1 levels [38]. We found that *Mrp1* was not expressed in hepatocytes, but was expressed in mHSCs. Activation of AHR directly regulated *Mrp1* expression in mHSCs, leading to decreased antioxidant capacity through increased GSH efflux and promoting ferroptosis specifically in mHSCs, without causing hepatocytes ferroptosis. Thus, targeting the mHSC‐specific ferroptosis pathway associated with high *Mrp1* expression holds promise as a potential therapeutic approach for the treatment of liver fibrosis.

When the intracellular GSH level falls below a critical threshold, GPX4 loses its function and can lead to the accumulation of ROS and cell death through iron‐dependent nonapoptotic mechanisms [39, 40]. Our findings suggest that GSH efflux plays a significant role in the regulation of ferroptosis sensitivity. AHR activation directly regulates *Mrp1* expression in mHSCs, diminishing their antioxidant capacity, and promoting ferroptosis by facilitating GSH efflux.

GPX4 is a central regulator of ferroptosis [41], and its activity depends on a certain threshold of GSH content [16]. Our study revealed that YH439 increased the expression of Mrp1 in mHSCs, facilitating GSH efflux. Although elevated levels of *Gpx4* were observed in mHSCs, the intracellular GSH content remained insufficient because of GSH efflux, thereby preventing *Gpx4* from exerting its antioxidant activity and promoting ferroptosis in mHSCs. *Mrp1* was not expressed in normal mouse hepatocytes, and there was no change in GSH content after YH439 treatment. Furthermore, increased expression of *Gpx4* improved the antioxidant capacity of normal mouse hepatocytes. This may explain why AHR alleviates liver fibrosis in mice by promoting ferroptosis in mHSCs without causing hepatocyte ferroptosis.

Ferroptosis, a recently discovered form of regulated cell death caused by iron‐dependent lipid peroxidation, differs substantially from other known types of cell death [20]. Erastin reduces liver fibrosis by modulating mHSC ferroptosis [26]. Consistent with previous studies, we found that AHR significantly alleviates CCL_4_‐induced liver fibrosis by inducing ferroptosis in mHSCs. A deeper understanding of the role of ferroptosis in the development of liver fibrosis will provide new perspectives for the diagnosis and treatment of this disease. Although the sensitivity of liver cells to ferroptosis inducers varies, targeting HSC‐specific ferroptosis is a direction for future research. Despite providing new perspectives on the treatment and prevention of liver fibrosis, the exact role of ferroptosis in this disease remains unclear and requires further investigation.

In conclusion, we demonstrated that AHR is an important transcription factor in liver fibrosis. Our findings suggest that AHR may represent a promising target for the prevention and treatment of liver fibrosis.

## Author Contributions

**Shenghui Liu:** writing – original draft (lead).

## Conflicts of Interest

The author declares no conflicts of interest.

## Supporting information


Data S1.

## Data Availability

The data that support the findings of this study are available from the corresponding author upon reasonable request.
