# Supplementary material for: Aryl Hydrocarbon Receptor Alleviates Hepatic Fibrosis by Inducing Hepatic Stellate Cell Ferroptosis
Source: J Cell Mol Med. 2024 Dec 9;28(23):e70278. doi: 10.1111/jcmm.70278 (PMC11628353; doi:10.1111/jcmm.70278)

**Aryl hydrocarbon receptor alleviates hepatic fibrosis by inducing hepatic stellate cell ferroptosis**

Shenghui Liu**^1^***

**^1^**Lin He’s Academician Workstation of New Medicine and Clinical Translation, Jining Medical University, Jining, China

***Correspondence**

Shenghui Liu, Lin He’s Academician Workstation of New Medicine and Clinical Translation, Jining Medical University, Jining, China,

Email: 1185553260@qq.com

**Supporting information**


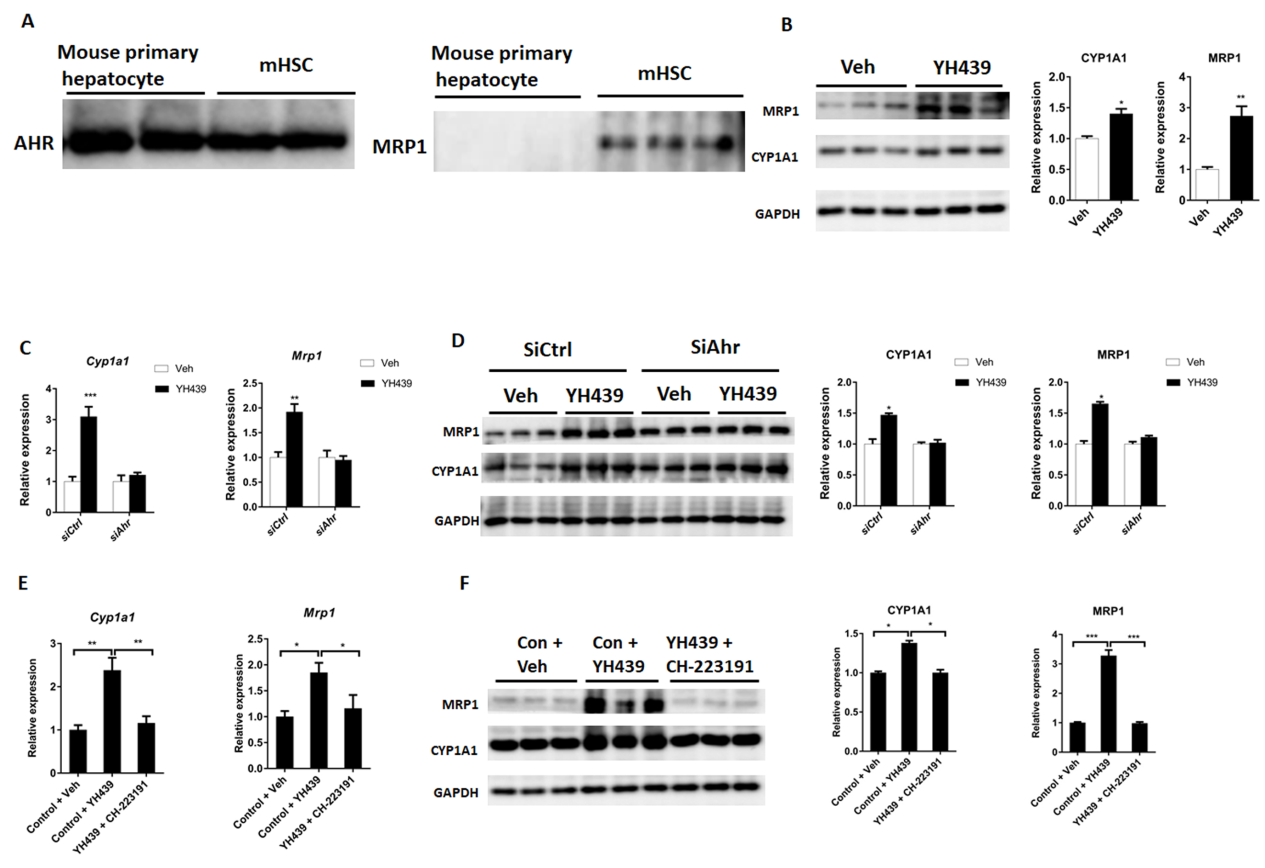


Figure S1. AHR promotes the expression of Mrp1 in mHSC. (A) The expression of AHR and MRP1 was detected by Western Blot. (B, D and F) The expression of CYP1A1 or MRP1 was detected by Western Blot in mHSC. (C, E) The expression of Cyp1a1 or Mrp1 was detected by QPCR in mHSC. Data are expressed as means ± SD; *p < 0.05, **p < 0.01 and ***p < 0.001; Student’s t test or one-way ANOVA.


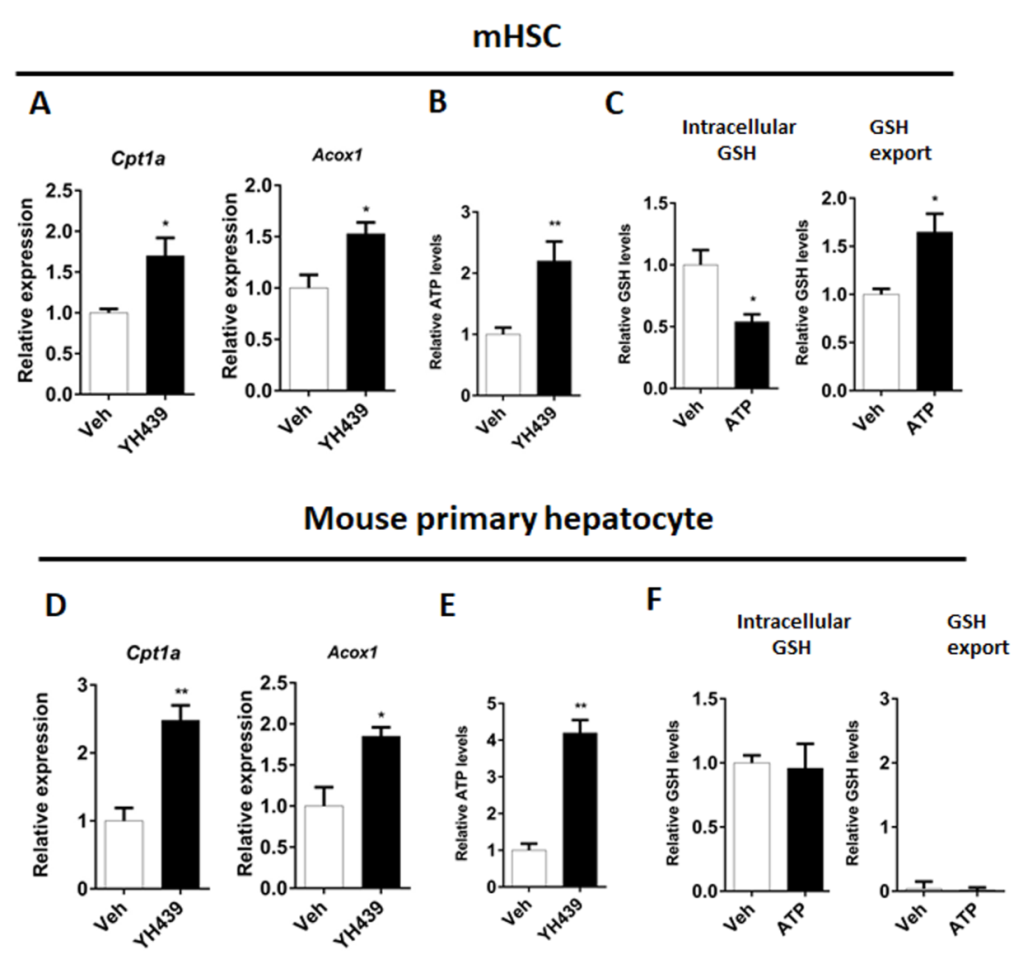
Figure S2. AHR increases GSH efflux by promoting mitochondrial oxidative phosphorylation (A) The expression changes of Cpt1a and Acox1 were detected by QPCR. (B) The change of intracellular ATP content was analyzed. (C) Changes in intracellular and extracellular GSH levels were analyzed. (D) The expression changes of Cpt1a and Acox1 were detected by QPCR. (E) The change of intracellular ATP content was analyzed. (F) Analysis of intracellular and extracellular GSH content changes. Data are expressed as means ± SD; *p < 0.05 and **p < 0.01; Student’s t test.


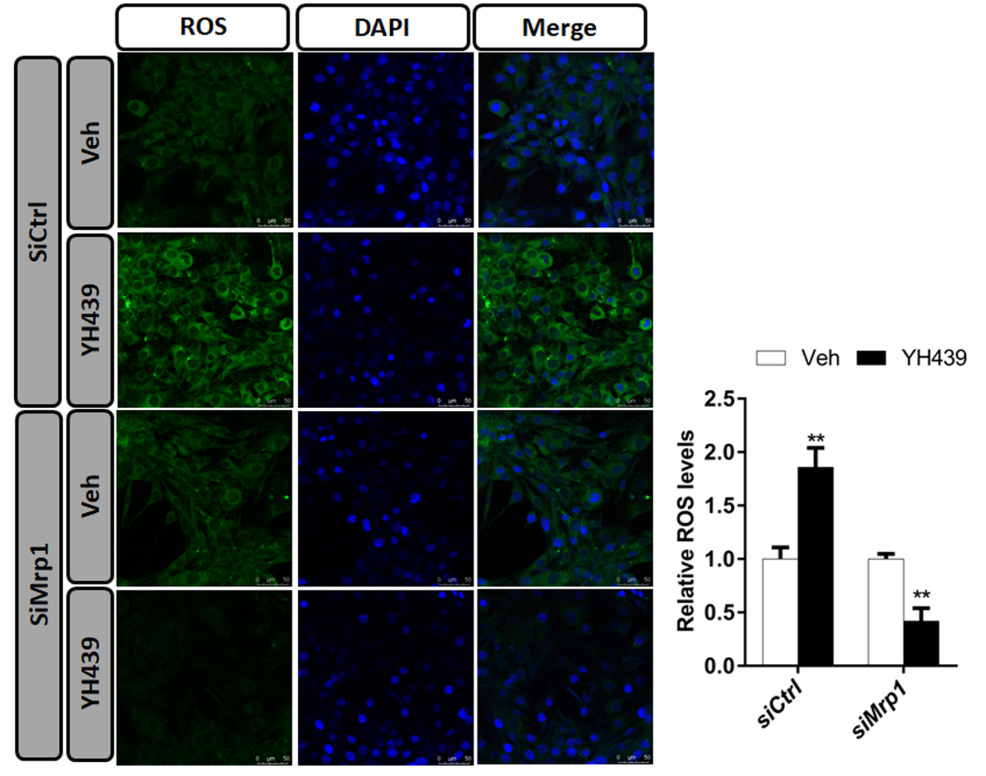

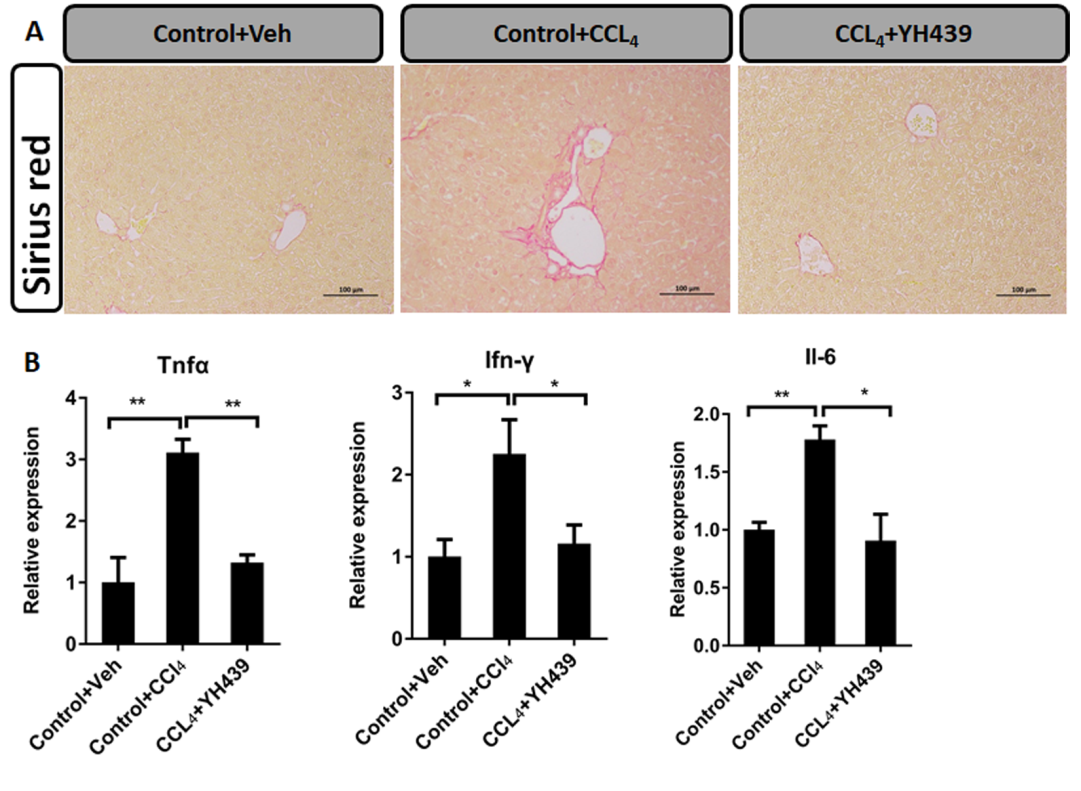
Figure S3. AHR promotes iron death in mHSC. H2DCFDA fluorescent probe was used to analyze the change of ROS content in mHSCs. The change of ROS content in mHSCs was quantitatively analyzed. Data are expressed as means ± SD; *p < 0.05 and **p < 0.01; Student’s t test. Scale bar =50 μm.

Figure S4. AHR alleviates liver fibrosis. (A) Sirius red staining. Scale bar =100μm. (B) Expressions of Tnfα, Ifn-γ and Il-6 were detected by QPCR. Data
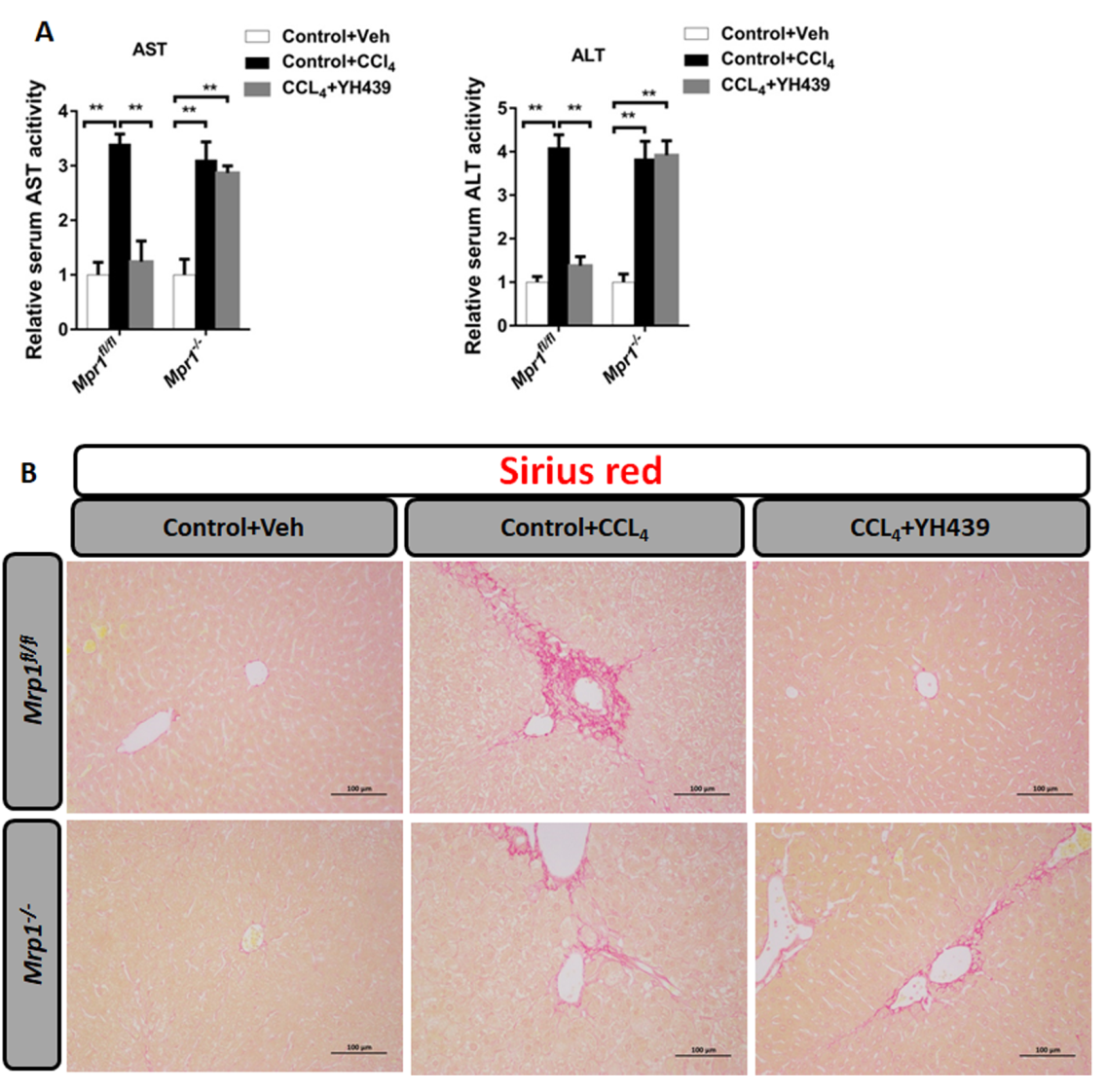
are expressed as means ± SD; *p < 0.05 and **p < 0.01; one-way ANOVA.

Figure S5. AHR alleviates liver fibrosis is abolished in HSC-specific Mrp1 deficient (Mrp1-/-) mice. (A) The changes of ALT and AST levels were detected (n=5). (B) Sirius red staining. Scale bar =100μm. Data are expressed as means ± SD; *p < 0.05 and **p < 0.01; one-way ANOVA.

Table S1. The Oligonucleotide Sequences for QPCR, EMSA, CHIP, Plasmid Construction or SiRNA-MRP1 related to Materials and methods.

| Names | Application | Sequence (5’-3’) |
| --- | --- | --- |
| M-*36B4*-F | QPCR | TGGAGACAAGGTGGGAGCC |
| M-*36B4*-R | QPCR | CACAGACAATGCCAGGACGC |
| M-*Mrp1*-F | QPCR | CCCTGTTCTCTGAAACTGTC |
| M-*Mrp1*-R | QPCR | TTCTTCTGATGTGTCCTCCT |
| M –*Cyp1a1*-F | QPCR | GTGAGCAAGGAGGCTAACTA |
| M –*Cyp1a1*-R | QPCR | CAAAGCATATGGCACAGATG |
| M -*Acox1*-F | QPCR | CTGAAATCAAGAGAAGCGAG |
| M –*Acox1*-R | QPCR | GAGAAAGTGGAAGGCATAGG |
| M –*Gpx4*-F | QPCR | GCAAAACTGACGTAAACTAC |
| M –*Gpx4*-R | QPCR | GCTCCTGCCTCCCAAACTGG |
| M –*Acsl4*-F | QPCR | TACTCTTCACCCCTTACACT |
| M -*Acsl4*-R | QPCR | AACCTTGCTCATAACATTCT |
| M –*Ptgs2*-F | QPCR | GACCAGAGCAGAGAGATGAA |
| M- *Ptgs2*-R | QPCR | TGAGGAGAACAGATGGGATT |
| M-*Nox1*-F | QPCR | ATCCTGATTCCTGTGTGTCG |
| M-*Nox1*-R | QPCR | GGCTTCTTCTGTAGCGTTCG |
| M- *Fth1*-F | QPCR | GTTATTTTGACCGAGATGAT |
| M- *Fth1*-R | QPCR | TTTGTGCAGTTCCAGTAGTG |
| M-*αSma-* F | QPCR | GGCATCCACGAAACCACCTA |
| M-*αSma-* R | QPCR | AGCCACCGATCCAGACAGAG |
| M-*Col1a1-* F | QPCR | CTGACTGGAAGAGCGGAGAG |
| M- *Col1a1-* R | QPCR | GACGGCTGAGTAGGGAACAC |
| M- *Col1a2-* F | QPCR | CCCAACCTGTAAACACCCCA |
| M- *Col1a2-* R | QPCR | CAAGCTGCCCGTCTCCTCAT |
| M- Il-6-F | QPCR | AGACTTCCATCCAGTTGCCT |
| M- Il-6-R | QPCR | TCTCATTTCCACGATTTCCC |
| M- Ifnγ-F | QPCR | TAGATGTGGAAGAAAAGAGT |
| M- Ifnγ-R | QPCR | TTGCTGAAGAAGGTAGTAAT |
| M- Tnfα-F | QPCR | AGACCCTCACACTCACAAAC |
| M- Tnfα-R | QPCR | CAATGACTCCAAAGTAGACC |
| XREL1-probe-F(Labeled) | EMSA | GCCCTAAAATCACGCAAGCACCGAG |
| XREL1-probe-R(Labeled) | EMSA | CTCGGTGCTTGCGTGATTTTAGGGC |
| XREL1-probe-F(Mut) | EMSA | GCCCTAAAATTCTTTAAGCACCGAG |
| XREL1-probe-R(Mut) | EMSA | CTCGGTGCTTAAAGAATTTTAGGGC |
| XREL1- probe-F(Cold) | EMSA | GCCCTAAAATCACGCAAGCACCGAG |
| XREL1- probe-R(Cold) | EMSA | CTCGGTGCTTGCGTGATTTTAGGGC |
| XREL2-probe-F(Labeled) | EMSA | TGCTCGCTCCCACGCGCGGGGCGGG |
| XREL2-probe-R(Labeled) | EMSA | CCCGCCCCGCGCGTGGGAGCGAGCA |
| XREL2-probe-F(Mut) | EMSA | TGCTCGCTCCTCTTTGCGGGGCGGG |
| XREL2-probe-R(Mut) | EMSA | CCCGCCCCGCAAAGAGGAGCGAGCA |
| XREL2- probe-F(Cold) | EMSA | TGCTCGCTCCCACGCGCGGGGCGGG |
| XREL2- probe-R(Cold) | EMSA | CCCGCCCCGCGCGTGGGAGCGAGCA |
| M-XREL1-CHIP-F | ChIP | CACGCAAGCACCGAGCGTTCTC |
| M-XREL1-CHIP -R | ChIP | CCGACACGGCCCGGAAGAACCC |
| M-XREL2-CHIP-F | ChIP | GTGGCCGTGCTCGCTCCCACGC |
| M-XREL2-CHIP -R | ChIP | CCGGCTGAGCGAGGAGGCGCAG |
| M-Mrp1-PGL3-F | Plasmid Construction | GGGTACCCCATATCCCTCCAGGTGCAACTG |
| M-Mrp1- PGL3-R | Plasmid Construction | CAAGCTTGCCAAGCTTGGCACGTCCTCCGA |
| M- Mrp1-PGL3-Mut1-F | Plasmid Construction | GCCCTAAAATTCTTTAAGCACCGAG |
| M- Mrp1-PGL3-Mut1-R | Plasmid Construction | CTCGGTGCTTAAAGAATTTTAGGGC |
| M- Mrp1-PGL3-Mut2-F | Plasmid Construction | GCTCGCTCCTCTTTGCGGGGCGGG |
| M- Mrp1-PGL3-Mut2-R | Plasmid Construction | CCCGCCCCGCAAAGAGGAGCGAGC |
| M-SiRNA-Mrp1 sense | SiRNA | ACUUCUUUCCCAGAAAGAGUA |
| M-SiRNA-Mrp1 antisense | SiRNA | CUCUUUCUGGGAAAGAAGUCA |
| M-SiRNA-Ahr sense | SiRNA | CGCAAGAUGUUAUUAAUAAGC |
| M-SiRNA-Ahr antisense | SiRNA | UUAUUAAUAACAUCUUGCGGG |
| Negative control sense | SiRNA | UUCUCCGAACGUGUCACGUTT |
| Negative control antisense | SiRNA | ACGUGACACGUUCGGAGAATT |

M: Mouse F: Forward R: Reverse


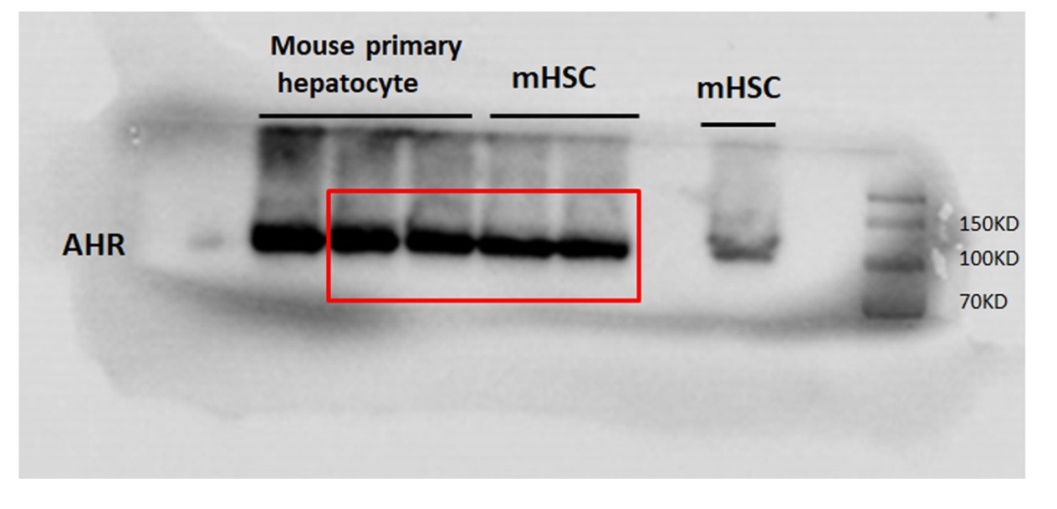
**Original data of Western blot**, **related to Figure S1A-1**

**Original data of Western blot**, **related to Figure S1A-2**


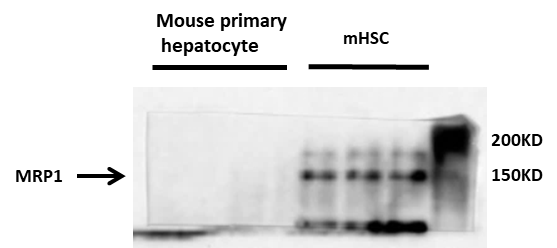


**Original data of Western blot**, **related to Figure S1B**


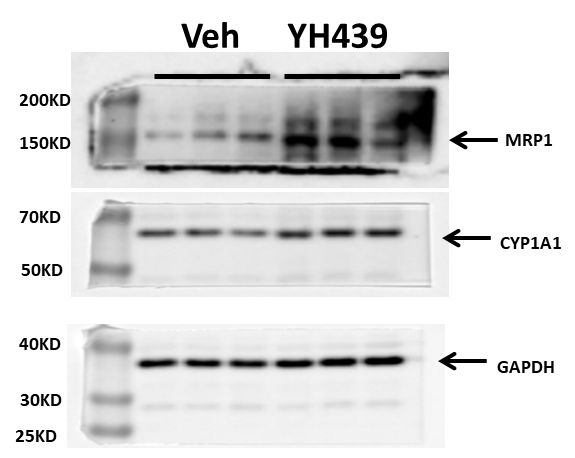


**Original data of Western blot**, **related to Figure S1D**

**
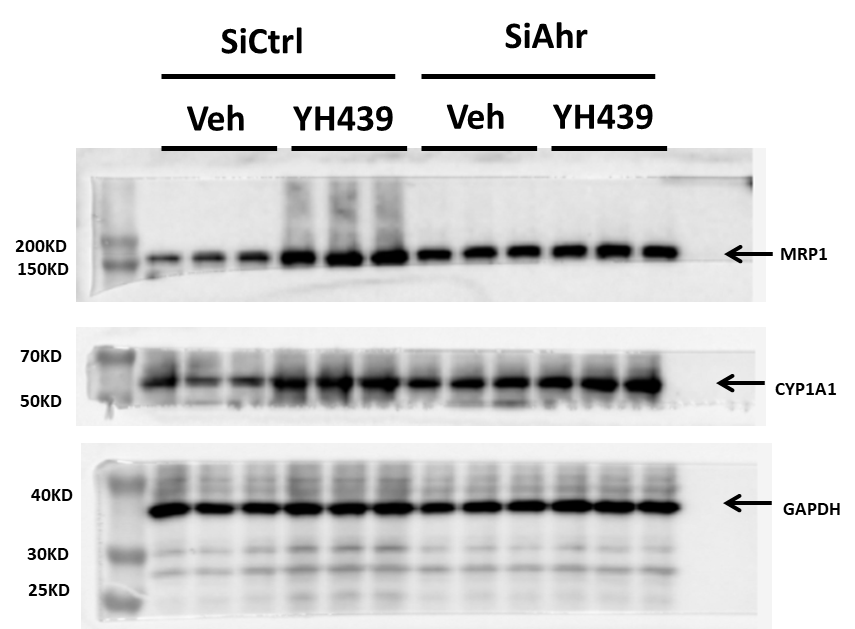
**

**Original data of Western blot**, **related to Figure S1F**


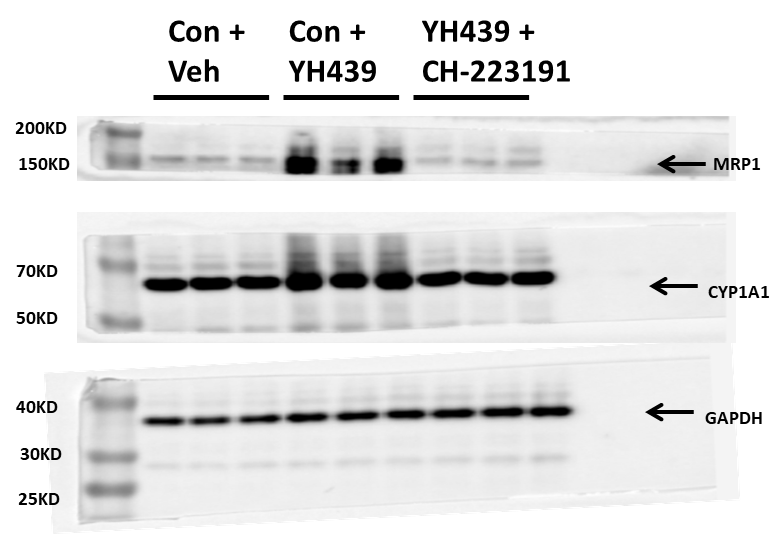

Supplement: Supplementary file 1 — Data S1. [file JCMM-28-e70278-s001.docx]
